# Growth performance and ultrasonographic body composition responses in lambs receiving Spirulina

**DOI:** 10.1007/s11250-026-05015-8

**Published:** 2026-04-16

**Authors:** Nadia Hamdi Fahim, Emad Mohammed Elken, Mahmoud A. Othman, Eltaher Mohammed Saudi, Reda Mohamed El-awady, Ahmed Sobhy Abdeltawab, Mohammed Hamed Eldawy

**Affiliations:** 1 Animal Production Department, Faculty of Agriculture, Al-Azhar University, Nasr City, Cairo Egypt; 2 Environment and Bio Agriculture Department, Faculty of Agriculture, Al-Azhar University, Cairo, Egypt; 3 Theriogenology Department, Faculty of Veterinary Medicine, Cairo University, Giza, Egypt; 4 Animal Production Department, Faculty of Agriculture, Cairo University, Giza, Egypt

**Keywords:** Spirulina, Lamb growth, Muscle and fat deposition, Ultrasonography

## Abstract

This study investigated the effects of oral Spirulina (*Arthrospira platensis*) administration on growth performance, rumen fermentation, blood biochemical parameters, and in vivo body composition in growing Barki lambs. Thirty male lambs (1–1.2 years old; 42.2 ± 1.9 kg) were randomly assigned to a control group fed the basal diet or two treatment groups receiving the basal diet with Spirulina at 0.1 (SPP1) or 0.2 g/kg body weight (SPP2) for 60 days. Longitudinal ultrasonography was applied to monitor changes in the *longissimus dorsi* muscle and fat deposition. Spirulina administration improved growth performance in a dose-dependent manner (*p* < 0.05). Rumen fermentation was enhanced in SPP2 lambs, as indicated by higher total volatile fatty acids and lower ammonia-N levels (*p* < 0.05). Blood biochemical analysis showed increased total protein concentrations and antioxidant capacity, with reduced urea and glucose levels in SPP2 lambs (*p* < 0.05). Ultrasound measurements showed dose-dependent increases in *longissimus dorsi* muscle area, perimeter, depth, and compactness, as well as backfat thickness, intramuscular fat, and fat-to-muscle ratio (*p* < 0.01). In conclusion, oral Spirulina at 0.2 g/kg BW enhanced growth performance, blood biochemical indicators, and in vivo body composition. Integrating Spirulina administration with longitudinal ultrasonography may provide a practical, non-invasive approach to monitor tissue development in small ruminants.

## Introduction

The global demand for red meat continues to rise (Komarek et al. 2021). Small ruminants are an efficient and effective option for red meat production in semi-arid/subtropical regions (Akinmoladun et al. 2019; Bhateshwar et al. 2022). The Barki sheep is one of the principal breeds with remarkable adaptability to the harsh desert conditions of the Mediterranean zone (Abousoliman et al. 2020; Aboulnaga and Abdelsabour 2023). Its high resilience enables efficient production of lean meat under challenging environments where many exotic breeds fail to perform, making Barki sheep a valuable genetic resource for meat production in desert and semi-arid regions (Aboul-Naga et al. 2025).

Improving growth performance in small ruminants under such conditions requires innovative nutritional and management strategies that support both productivity and sustainability (Kadim et al. 2025). One promising approach is the use of functional feed additives. Among these additives, Spirulina (*Arthrospira platensis*) is an exemplary microalga in terms of protein, vitamins, essential fatty acids, and antioxidant compounds (Anvar and Nowruzi 2021). It is considered a ‘superfood’ due to its high nutritional value compared to other plant sources (Priyanka et al. 2023). It has been demonstrated that spirulina has promising effects on enhancing growth rate and blood biochemistry parameters in small ruminants (Irshad et al. 2024; Firdaus et al. 2025). Most studies, however, have focused on endpoint measures of carcass composition or biochemical reactions, rather than on capturing dynamic in vivo alterations in muscle and fat deposition over time (Schumacher et al. 2022).

Ultrasonography has become a vital non-invasive tool for monitoring livestock, enabling dynamic in vivo assessment of tissue structure with high correlations to meat quality indicators (Scholz et al. 2015; Lazar et al. 2023). Ultrasonography avoids slaughter, enables real-time data collection, reduces handling stress, and therefore meets the sustainability and ethical requirements of production. The method reduces sampling in the terminal phase while allowing regular appraisal of production traits. Besides, ultrasound scanning can be used to select lamb meat and predict potential market value (Wuliji et al. 2024). Ultrasonography has not been widely used in nutritional trials to continue tissue development phenotyping, despite its proven effectiveness. This integration may help close the gap between precision-based animal management and traditional feed trials. This study addresses these gaps by integrating real-time ultrasonography with Spirulina administration to monitor dynamic changes in muscle and fat deposition in lambs.

Therefore, the study aimed to (i) examine the dose-dependent effects of oral Spirulina treatment (0.1 and 0.2 g/kg BW) on growth performance, rumen fermentation, and body composition as measured using real-time ultrasonography in growing Barki lambs; and (ii) investigate an integrated approach that combines sustainable nutrition with precision phenotyping to provide a welfare-compatible and management-oriented system for monitoring tissue development, thereby improving productivity in small ruminant production systems.

## Materials and methods

### Ethical approval

This study was approved by the Institutional Animal Care and Use Committee (CU-IACUC), Cairo University, Egypt (No. CU II F 20 25).

### Animals and management

The experiment was conducted at the Experimental and Research Farm, Faculty of Agriculture, Al-Azhar University, Cairo, Egypt (30°03′14″N, 31°19′04″E). The lambs’ clinical health status was confirmed prior to the start of the trial. All lambs underwent a visual inspection and a veterinary clinical examination to ensure they were free of apparent disease, physical abnormalities, or physiological disorders before inclusion in the experiment. Based on farm records, thirty clinically healthy male Barki lambs (1-1.2 years old; 42.2 ± 1.9 kg) were selected to be included in the experiment. Lambs’ groups were housed under the same environmental conditions, with ambient temperature ranging from 14 to 29 °C during the experimental period and relative humidity averaging approximately 60%, under a natural 12 h light/dark cycle. Experimental lambs were fed the same balanced basal diet formulated according to NRC (2007) recommendations. The concentrated feed included 70% TDN, 14% crude protein, 8% fiber, 4% ether extract, and 4% ash. It was composed of 51% yellow corn, 24% wheat bran, 12% cottonseed cake, 6% soybean meal, 1.5% ground limestone, 5% common salt, and 0.5% mineral-vitamin premix. All lambs received the same management with free access to fresh water.

### Experimental design and treatments

Lambs were randomly allocated into three equal groups (*n* = 10 per group) using a computer-generated randomization procedure. The stocking density was kept at 2.5 m² per animal, allowing ample pens for natural mobility and contact. Each pen was 5 m × 5 m, totaling 25 m². The assigned groups were: control (basal diet only), SPP1 (basal diet + Spirulina at 0.1 g/kg body weight), and SPP2 (basal diet + Spirulina at 0.2 g/kg body weight). The experimental period lasted for 60 days.

### Spirulina preparation and oral administration

Spirulina used in this study was prepared in the research laboratory and analyzed for its proximate composition (Table 1). Spirulina powder was freshly dissolved in distilled water (1 g/10 mL) before administration. Treated lambs were given the solution orally once daily using a calibrated drench syringe. Administration was performed before the morning feeding to ensure complete consumption and minimize dose variability among animals (Bowman and Sowell 1997). The method was applied by well-trained personnel to reduce stress on the animals.

**Table 1:** Chemical composition of Spirulina (*Arthrospira platensis*)

Composition Per 100 g	Values
Moisture %	7.2
Crude protein%	58.4
Total carbohydrate%	10.4
Total lipids (ether extract) %	5
Fibers %	6.02
Ash%	8.9
Carotenes (mg/g)	0.22
Chlorophyll (mg/g)	1.2
Phycocyanin (mg/g)	2.3
Calcium (mg/g)	3.34
Iron (mg/g)	2.17
Phosphorus (mg/g)	11
Magnesium (mg/g)	5
Copper (mg/g)	0.67
Potassium (mg/g)	20
Sodium (mg/g)	10

### Growth measurements

Body weight was recorded on days 0, 20, 40, and 60 using a calibrated digital scale (± 0.01 kg). Lambs were weighed at the same time of day before the morning feeding. They were fasted prior to weighing to ensure consistent measurements. The weight gain was calculated as the difference between the final and initial body weight divided by the total number of experimental days (Saleem and Singer 2018).

### Blood sampling and biochemical analysis

The blood samples (10 ml) were taken from the jugular vein of the experimental lambs at 0, 30, and 60 days before morning feeding. The plasma was separated by centrifugation (3,000 g, 15 min, 4 °C) and stored at -20 °C. Total protein and albumin were determined using a commercial kit (Spectrum, Egyptian Company for Biotechnology, Egypt) and measured colorimetrically at 546 nm, as described by Henry (1974). The difference between total protein and albumin is estimated as Globulin. Commercial diagnostic kits were used to determine total antioxidant capacity (Koracevic et al. 2001), Urea (Fawcett and Scott 1960), and Glucose (Badugu et al. 2006).

### Ultrasonographic evaluation

**Fig. 1:**
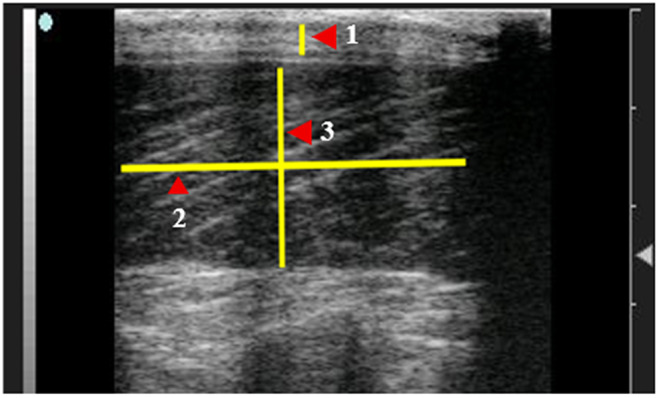
Measurements of the *Longissimus dorsi* muscle of Barki rams using ImageJ software.1 = Back fat thickness (BFT, mm), 2 = *Longissimus dorsi* muscle length (LDL, mm), 3 = *Longissimus dorsi* muscle depth (LDD, mm). Control=basal diet, SPP1 = basal diet + Spirulina at 0.1 g/kg BW, and SPP2 = basal diet + Spirulina at 0.2 g/kg BW

**Fig. 2:**
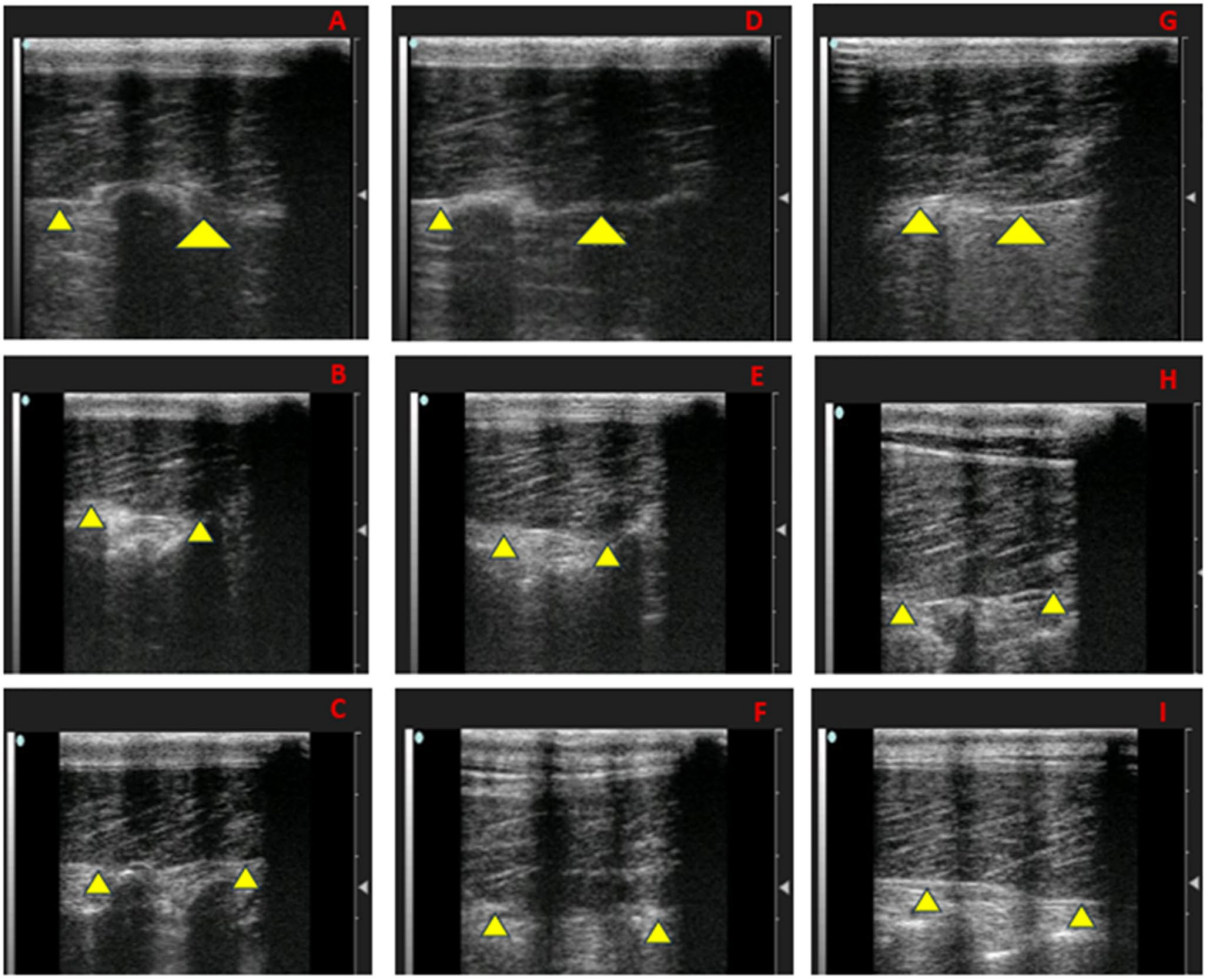
Echogenicity of the Spirulina effects on the *Longissimus dorsi* muscle (LDM) in Barki rams, as pointed with yellow arrows. Control at 0 day(**A**), 30 day (**B**), and 60 day (**C**). SPP1 at 0 day(**D**), 30 day (**E**), and 60 day (**F**). SPP2 at day 0 (**G**), 30 day (**H**), and 60 day (**I**). Control=basal diet, SPP1 = basal diet + Spirulina at 0.1 g/kg BW, and SPP2 = basal diet + Spirulina at 0.2 g/kg BW

**Fig. 3:**
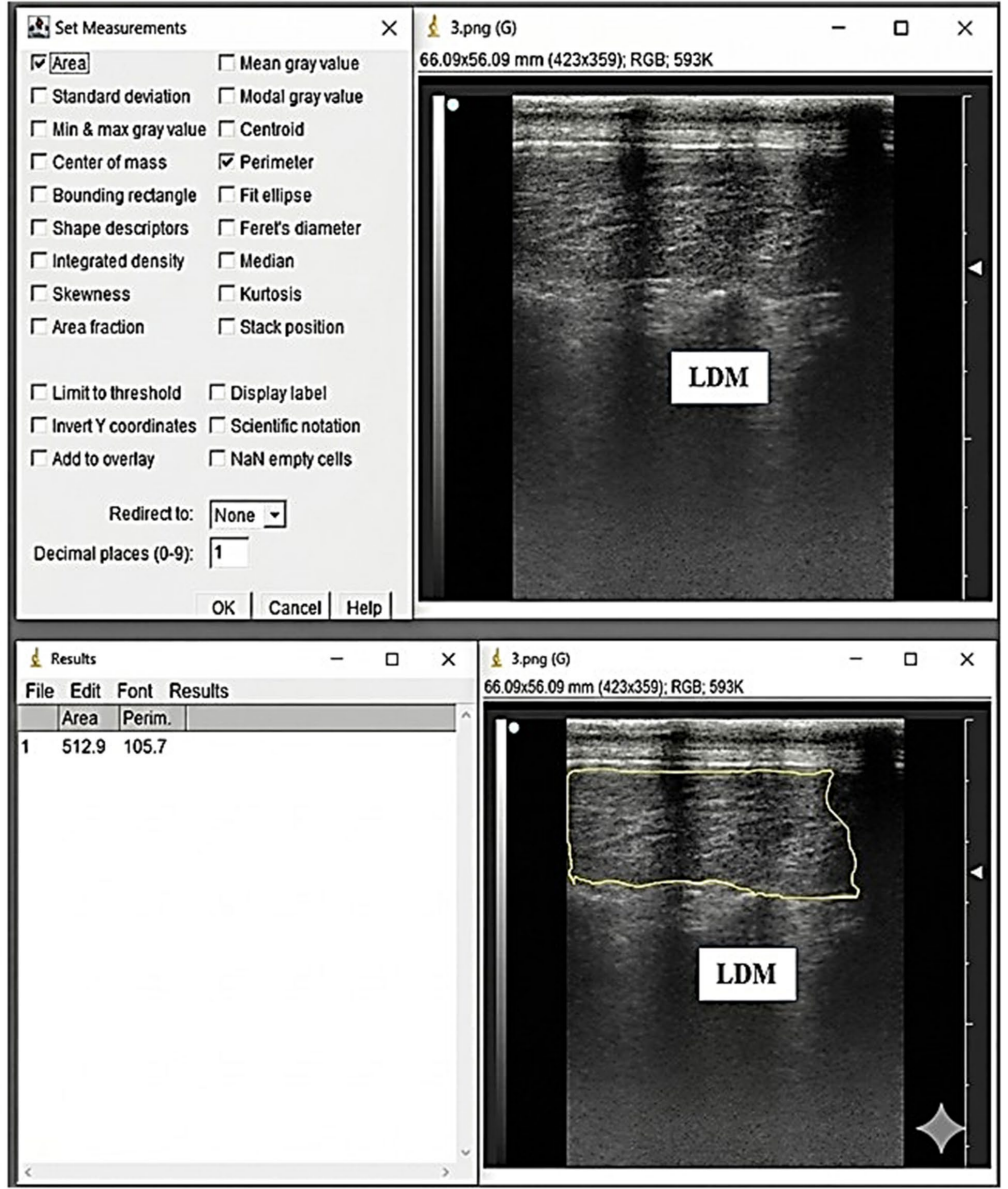
Measurements of the *Longissimus dorsi* muscle (LDM) of Barki rams using ImageJ software. 1 = Perimeter (mm) and 2 = *Area* (mm2). Control=basal diet, SPP1 = basal diet + Spirulina at 0.1 g/kg BW, and SPP2 = basal diet + Spirulina at 0.2 g/kg BW

It has been shown that ultrasonographic measurements of *longissimus dorsi* muscle and backfat are well correlated with direct carcass measurements in lambs (Esquivelzeta et al. 2012). This indicates that ultrasonography could be a reliable non‑invasive method for predicting carcass traits (Lazar et al. 2023). Real-time ultrasonography of the *longissimus dorsi* muscle and subcutaneous fat was performed at 6 cm depth using a 7.5 MHz linear probe (EXAGO, Echo Control Medical, France). Scans were taken on days 0, 30, and 60 at the 12th -13th thoracic vertebrae on the left side after shaving and applying acoustic gel. All settings (gain, contrast, depth) were standardized, and the same trained operator performed all scans to minimize measurement variability. Ultrasound Images were processed using ImageJ software (Schindelin et al. 2012) to determine muscle depth (LDMD, mm), muscle length (LDML, mm), and backfat thickness (BFT, mm), as illustrated in Fig. 1. Muscle echogenicity was evaluated by determining the mean gray value of the *longissimus dorsi* muscle (Fig. 2), which was used as an indicator of intramuscular fat deposition (Young et al. 2015). Additionally, muscle perimeter (mm) and cross-sectional area (mm²) were determined using ImageJ, as shown in Fig. 3.

Ultrasonographic measurements of *longissimus dorsi* muscle depth (LDMD), muscle length (LDML), and backfat thickness (BFT) were used to derive two comparative indices in this study:

- the Muscle Compactness Index (MCI) to capture changes in muscle shape and compactness. It was calculated as MCI = LDMD / LDML.
- the Fat-to-Muscle Ratio (FMR, %), to provide a simple comparative measure of fat relative to muscle. It was calculated as:

FMR (%) = (BFT / LDMD) × 100. Values were multiplied by 100 to express the ratio as a percentage, making it easier to compare between treatments.

Both derived indices were intended to allow comparisons among treatments and to track changes over time without implying absolute muscle or fat quantities. The use of ultrasonography to derive such an index has been validated in previous studies. Orman et al. (2008) demonstrated that ultrasound-based measurements of muscle and fat are closely correlated with carcass traits, supporting the reliability of derived indices for monitoring body composition in live lambs. Consistently, Pimentel et al. (2023) and Muñoz-Osorio et al. (2024) reported that relative indices obtained by ultrasonographic techniques were effective tools for monitoring changes in muscle development and fat deposition.

### Rumen fermentation parameters

A rumen fluid sample (100 mL) was collected aseptically using a flexible stomach tube 4 h after morning feeding. This timing allows the measurement of the postprandial peak in VFA production (Pless et al. 2018). The samples were filtered through cheesecloth and immediately analyzed for pH using a digital pH meter (Orion 680, Thermo Scientific, USA). Ammonia-N (NH_3_-N) and total volatile fatty acids (TVFAs) were determined following the procedures of Conway (1957) and Borhami et al. (1979), respectively.

### Statistical analysis

Data were analyzed using SPSS v25 (IBM Corp., Armonk, NY, USA). Normality and homogeneity of data were verified before analysis by the Shapiro-Wilk and Levene’s test (Shapiro and Wilk 1965). Repeated-measures data (body weight, blood metabolites, and ultrasonographic traits) were analyzed using a linear mixed-effects model with treatment, time, and their interaction as fixed effects, and individual lamb as a random effect. Single-time-point variables were analyzed using one-way ANOVA. Differences were considered significant at *p* < 0.05.

## Results

### Growth performance

**Fig. 4:**
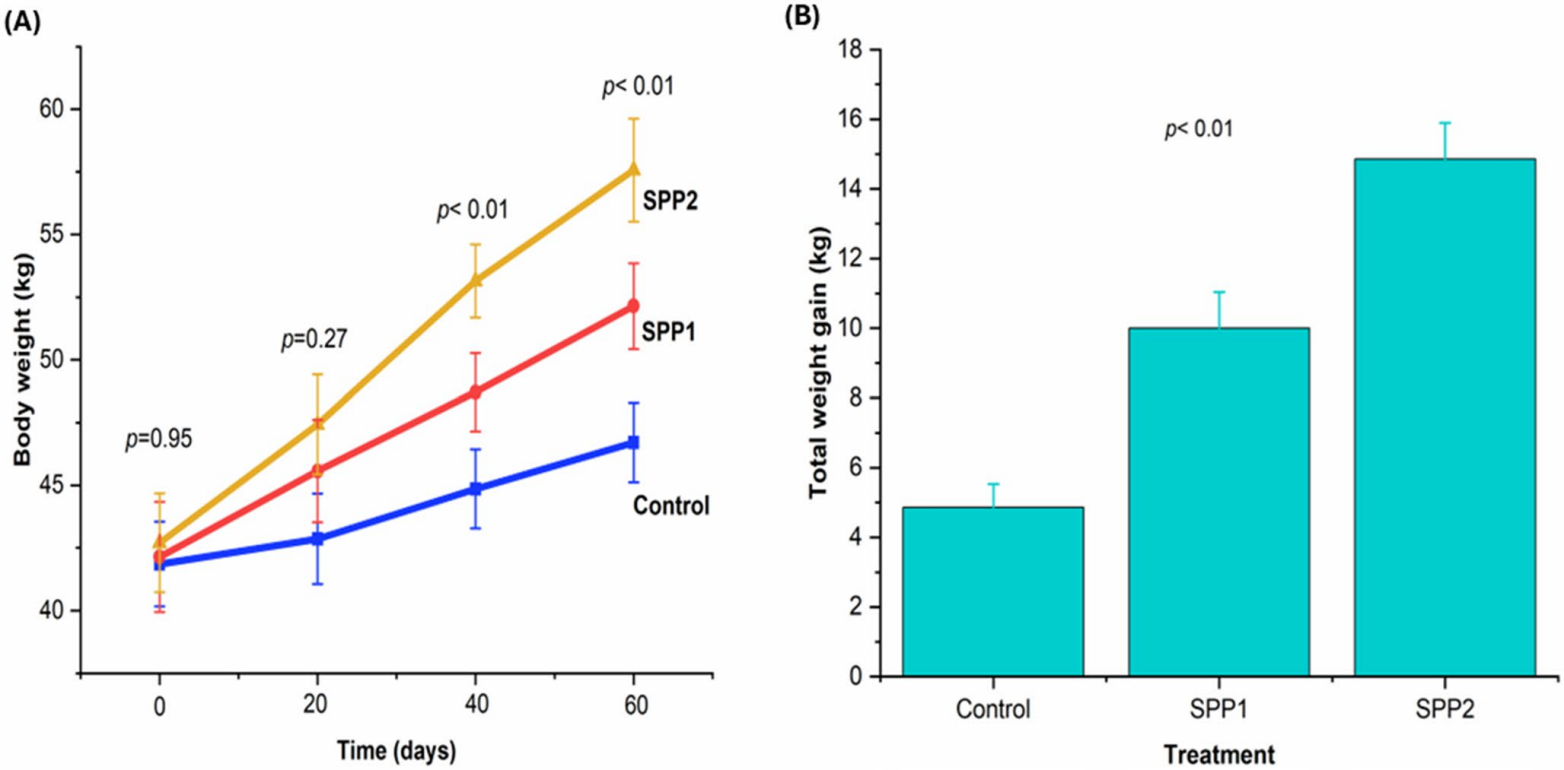
Effects of oral Spirulina administration on growth performance in Barki lambs. Control=basal diet, SPP1 = basal diet + Spirulina at 0.1 g/kg BW, and SPP2 = basal diet + Spirulina at 0.2 g/kg BW

Table 2 shows that Spirulina administration increased the final body weight and the weight gain of treated lambs (*p* < 0.01). Lambs of SPP2 showed the greatest increase in growth performance, followed by those of SPP1, compared to the control group. Figure 4 illustrates this trend, showing that these weights increased with higher doses of Spirulina administration.

**Table 2:** Growth performance of lambs administered Spirulina

Item	Treatments (mean ± SE)				
	Control	SPP1	SPP2		*p*-value
Initial weight, kg	41.86 ± 1.69	42.14 ± 2.19	42.71 ± 1.97		0.95
Final weight, kg	46.71^c^ ± 1.58	52.14^b^ ± 1.71	57.57^a^ ± 2.05		< 0.01
Weight gain, kg	4.86^c^ ± 0.67	10.00^b^ ± 1.04	14.86^a^ ± 1.03		< 0.01

Control= basal diet, SPP1 = basal diet + 0.1 g Spirulina/kg BW, SPP2 = basal diet + 0.2 g Spirulina /kg BW. Means ± SE represent least-squares means. Superscripts (a, b, c) within the same row indicate significant differences (*p* < 0.05, row-wise comparison)

### Rumen fermentation parameters

**Fig. 5:**
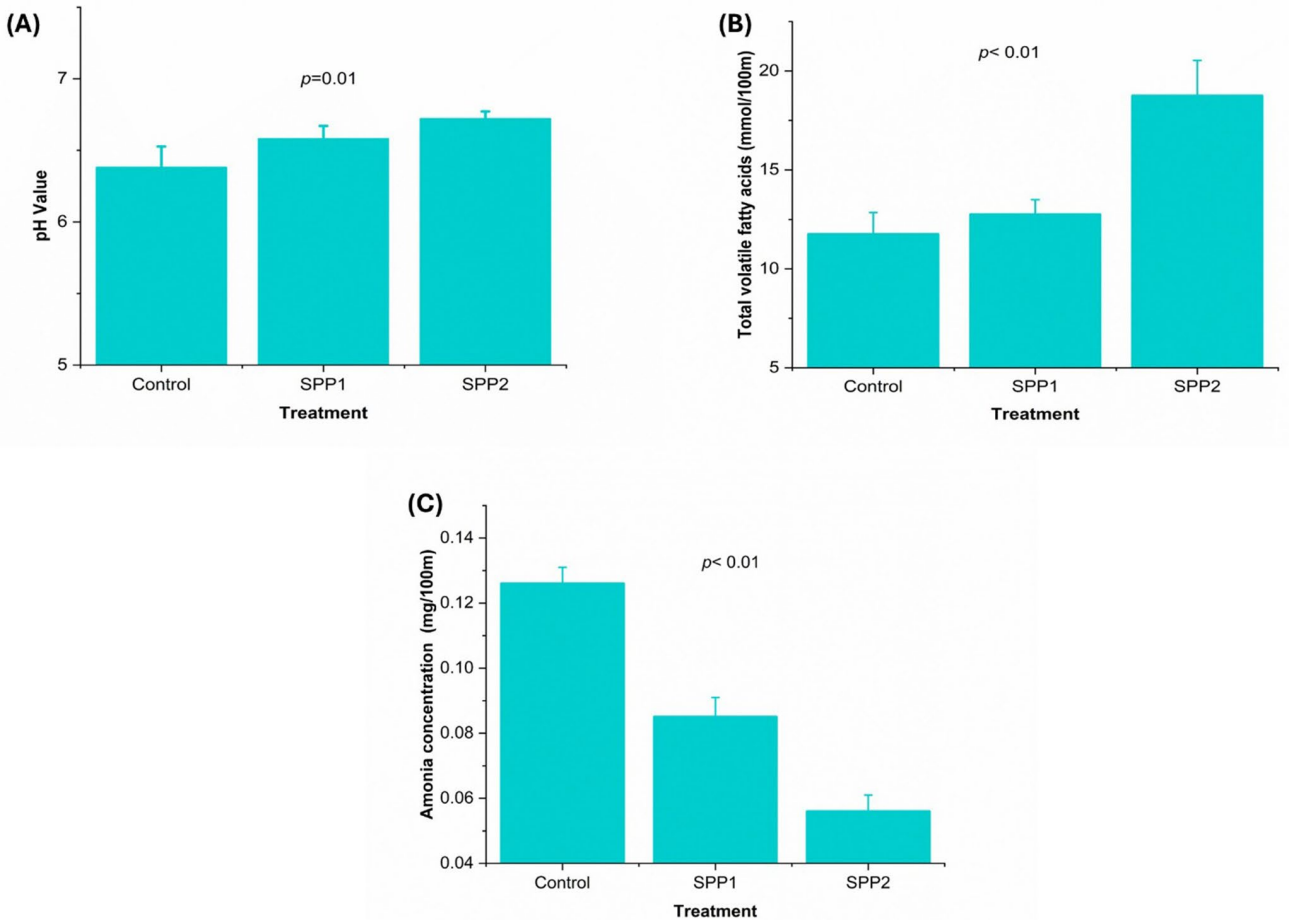
Effects of oral Spirulina administration on rumen fermentation traits in Barki lambs. Control=basal diet, SPP1 = basal diet + Spirulina at 0.1 g/kg BW, and SPP2 = basal diet + Spirulina at 0.2 g/kg BW

As presented in Fig. 5, ruminal pH increased slightly with increasing Spirulina inclusion (*p* = 0.09). TVFAs were higher while NH_3_-N was lower in SPP2 than in SPP1 and the control (*p* < 0.01).

### Blood biochemical profiles

All experimental lambs showed no significant differences in blood biochemical parameters (Table 3). After 30 days (Table 4), total protein, albumin concentrations, and total antioxidant capacity were significantly higher (*p* < 0.01) in SPP2 than in SPP1 and the control, while urea and glucose levels were reduced (*p* < 0.05) in a dose-dependent manner. At 60 days (Table 5), total protein and albumin remained elevated (*p* < 0.01) in SPP2, whereas urea and glucose concentrations decreased (*p* < 0.05) relative to control. Total antioxidant capacity increased (*p* = 0.02) in SPP2. Globulin content showed no significant variation (*p* > 0.05) among groups either at 30 days or 60 days of the experiment.

**Table 3:** Blood biochemical profiles of lambs in the start of the experiment

Item	Treatments (mean ± SE)			
	Control	SPP1	SPP2	*p*-value
Total protein (g/dL)	5.53 ± 0.116	5.42 ± 0.110	5.29 ± 0.420	0.41
Albumin (g/dL)	3.30 ± 0.081	3.28 ± 0.112	3.36 ± 0.110	0.84
Globulin (g/dL)	2.23 ± 0.113	2.14 ± 0.151	1.92 ± 0.090	0.21
Urea (mg/dL)	80.50 ± 2.86	77.18 ± 3.10	82.63 ± 3.91	0.52
Glucose (mg/dL)	71.38 ± 3.86	68.34 ± 4.09	66.37 ± 4.05	0.67
Total antioxidant (mmol/ml)	1.48 ± 0.171	1.55 ± 0.204	1.80 ± 0.278	0.56

Control= basal diet, SPP1 = basal diet + 0.1 g Spirulina/kg BW, SPP2 = basal diet + 0.2 g Spirulina /kg BW. Means ± SE represent least-squares means. No significant differences among groups (*p* < 0.05)

**Table 4:** Blood biochemical profiles of lambs administered spirulina after 30 days of the experiment

Item	Treatments (mean ± SE)			
	Control	SPP1	SPP2	*p*-value
Total protein (g/dL)	5.36^b^ ± 0.06	5.47^b^ ± 0.18	6.28^a^ ± 0.18	< 0.01
Albumin (g/dL)	3.01^b^ ± 0.07	3.06^b^ ± 0.05	3.66^a^ ± 0.12	< 0.01
Globulin (g/dL)	2.34 ± 0.10	2.35 ± 0.19	2.62 ± 0.12	0.32
Urea (mg/dL)	84.36^a^ ± 2.66	77.99^a^ ± 2.4	69.69^b^ ± 2.73	< 0.01
Glucose (mg/dL)	91.83^a^ ± 4.81	84.82^ab^ ± 3.45	76.92b ± 2.70	0.03
Total antioxidant (mmol/ml)	1.38^b^ ± 0.15	1.47^b^ ± 0.19	2.32a ± 0.26	< 0.01

Control= basal diet, SPP1 = basal diet + 0.1 g Spirulina/kg BW, SPP2 = basal diet + 0.2 g Spirulina /kg BW. Means ± SE represent least-squares means. Superscripts (a, b, ab) within the same row indicate significant differences (*p* < 0.05, row-wise comparison)

**Table 5:** Blood biochemical profiles of lambs administered Spirulina after 60 days of the experiment

Item	Treatments (mean ± SE)			
	Control	SPP1	SPP2	*p*-value
Total protein (g/dL)	5.48^c^ ± 0.24	6.11^b^ ± 0.19	6.84^a^ ± 0.17	< 0.01
Albumin (g/dL)	3.00^c^ ± 0.04	3.32^b^ ± 0.04	3.73^a^ ± 0.10	< 0.01
Globulin (g/dL)	2.47 ± 0.27	2.79 ± 0.20	3.10 ± 0.12	0.12
Urea (mg/dL)	90.25^a^ ± 1.28	83.95^ab^ ± 2.42	78.22^c^ ± 3.09	< 0.01
Glucose (mg/dL)	91.11^a^ ± 2.47	83.73^ab^ ± 2.82	80.07^c^ ± 2.71	0.02
Total antioxidant (mmol/ml)	1.33^b^ ± 0.14	1.81^ab^ ± 0.28	2.22^a^ ± 0.15	0.02

Control= basal diet, SPP1 = basal diet + 0.1 g Spirulina/kg BW, SPP2 = basal diet + 0.2 g Spirulina /kg BW. Means ± SE represent least-squares means. Superscripts (a, b, ab, c) within the same row indicate significant differences (*p* < 0.05, row-wise comparison)

### Ultrasonographic assessment of body composition traits

**Fig. 6:**
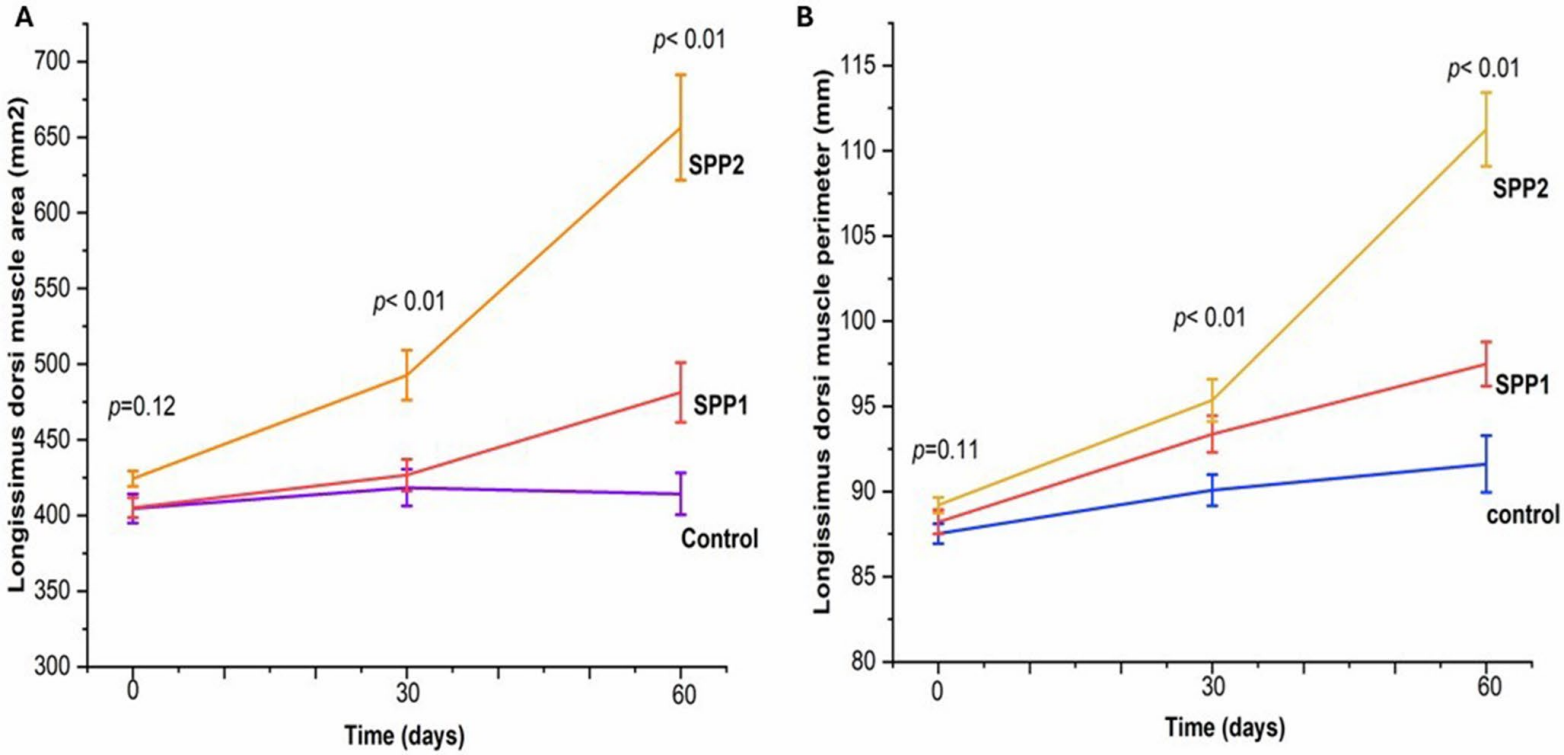
Effects of oral Spirulina administration on *Longissimus dorsi* muscle area and perimeter in Barki lambs. Control=basal diet, SPP1 = basal diet + Spirulina at 0.1 g/kg BW, and SPP2 = basal diet + Spirulina at 0.2 g/kg BW

**Fig. 7:**
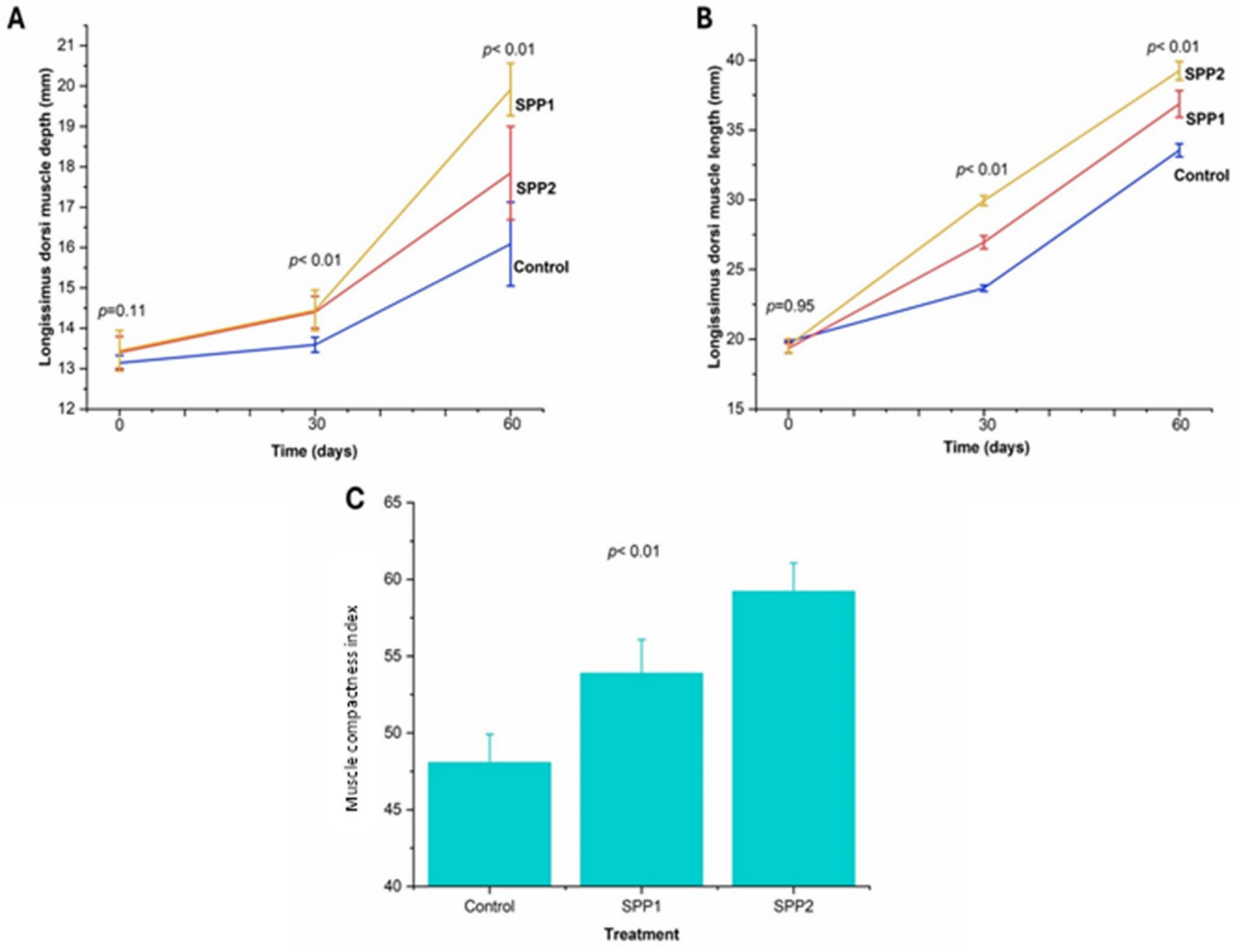
Effects of oral Spirulina administration on *longissimus dorsi* muscle (depth and length) and the muscle compactness index in Barki lambs. Control=basal diet, SPP1 = basal diet + Spirulina at 0.1 g/kg BW, and SPP2 = basal diet + Spirulina at 0.2 g/kg BW

**Fig. 8:**
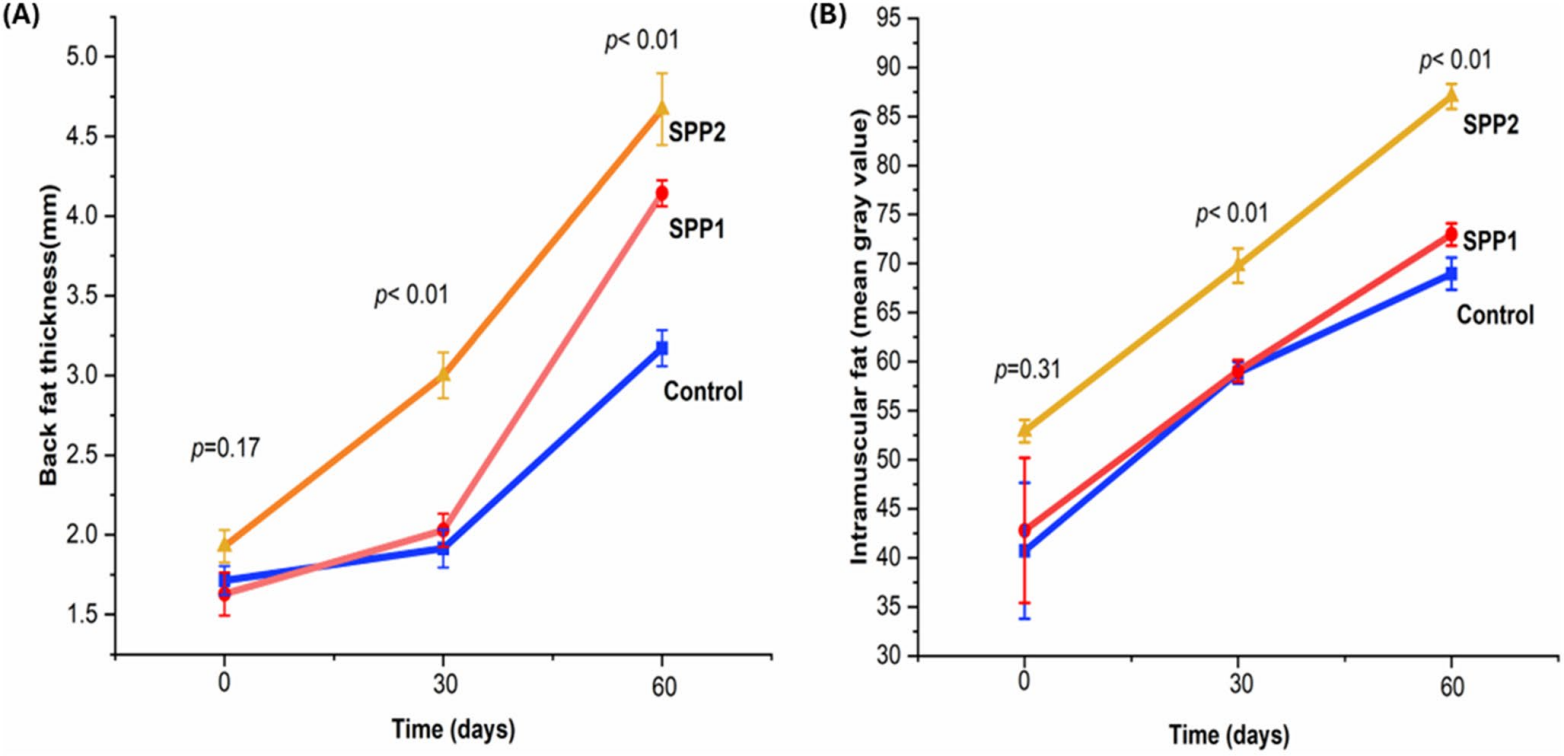
Effects of oral Spirulina administration on back fat thickness and intramuscular fat (marbling meat) in Barki lambs. Control=basal diet, SPP1 = basal diet + Spirulina at 0.1 g/kg BW, and SPP2 = basal diet + Spirulina at 0.2 g/kg BW

**Fig. 9:**
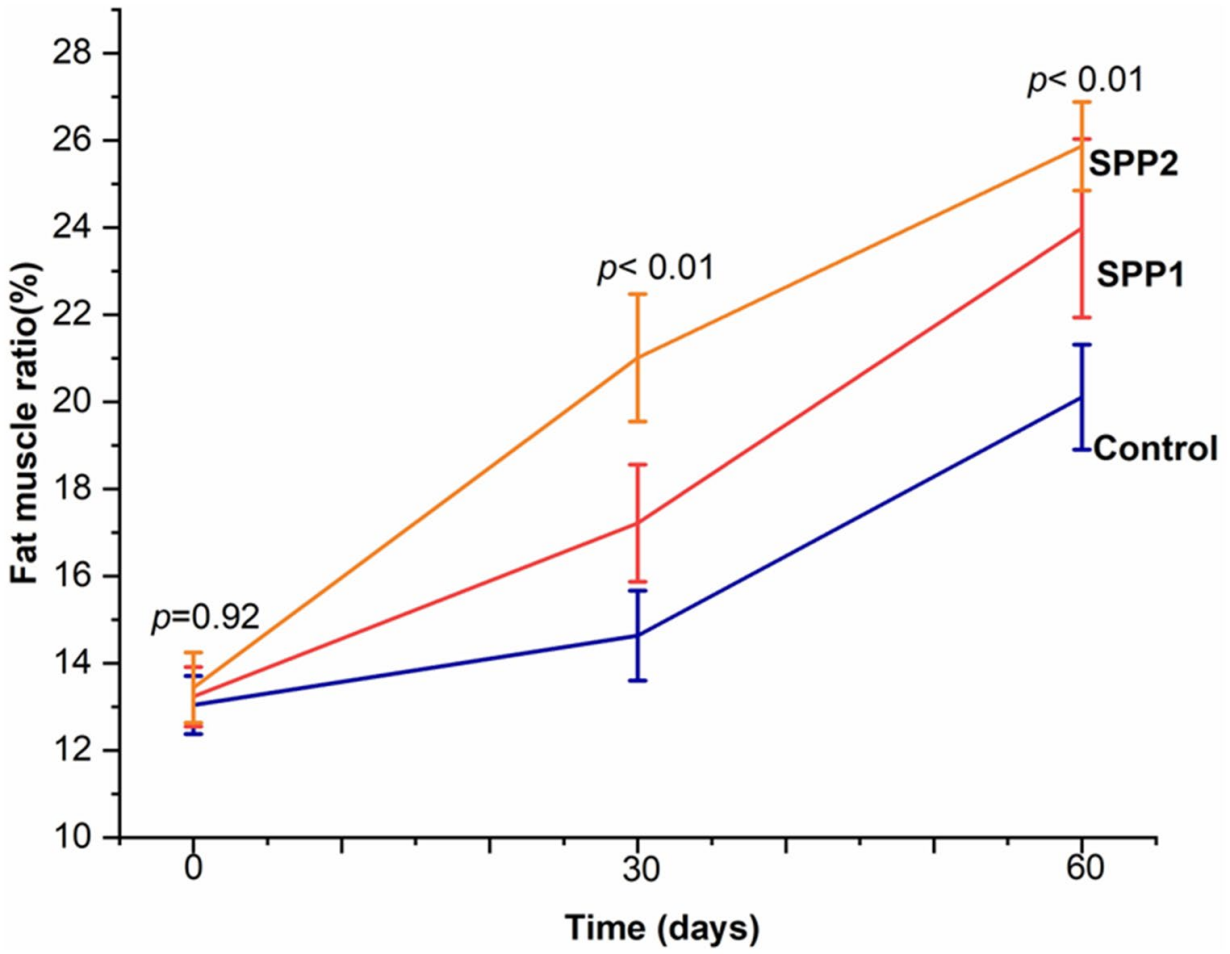
Effects of oral Spirulina administration on fat-to-muscle-ratio (%) in Barki lambs. Control=basal diet, SPP1 = basal diet + Spirulina at 0.1 g/kg BW, and SPP2 = basal diet + Spirulina at 0.2 g/kg BW

The experimental lambs had no significant differences in any ultrasonographic measurements (*p* > 0.05). After 30 and 60 days, the *longissimus dorsi* muscle area (Table 6) and perimeter (Table 7) increased significantly in Spirulina-administered lambs (*p* < 0.01). Figure 6 visualizes this trend, displaying a dose-dependent effect. Values of MCI increased over time in both control and Spirulina-treated groups. Higher Spirulina doses were significantly associated with higher MCI readings at later sampling points (Fig. 7). Moreover, backfat thickness, intramuscular fat indicators (Fig. 8), muscle depth, and muscle length (Fig. 8) increased significantly with increasing Spirulina administration level (*p* < 0.01). In addition, the fat-to-muscle ratio increased over time in all groups and showed a significant dose-dependent effect of Spirulina administration (Table 8; Fig. 9).

**Table 6:** *Longissimus dorsi* muscle area (mm^2^) in lambs administered Spirulina

Time (days)	Treatments (mean ± SE)			
	Control	SPP1	SPP2	*p*-value
0	404.57 ± 9.54	405.11 ± 6.59	424.34 ± 5.03	0.95
30	418.38^c^ ± 12.24	426.58^b^ ± 10.38	492.67^a^ ± 16.42	< 0.01
60	414.24^c^ ± 13.87	481.28^b^ ± 19.73	656.42^a^ ± 34.79	< 0.01

Control= basal diet, SPP1 = basal diet + 0.1 g Spirulina/kg BW, SPP2 = basal diet + 0.2 g Spirulina /kg BW. Means ± SE represent least-squares means. Superscripts (a, b, c) within the same row indicate significant differences (*p* < 0.05, row-wise comparison)

**Table 7:** *Longissimus dorsi* muscle perimeter (mm) in lambs administered Spirulina

Time (days)	Treatments (mean ± SE)			
	Control	SPP1	SPP2	*p*-value
0	87.52 ± 0.59	88.21 ± 0.71	89.2 ± 0.46	0.95
30	90.07^c^ ± 0.91	93.37^b^ ± 1.07	95.35^a^ ± 1.25	< 0.01
60	91.61^c^ ± 1.67	97.48^b^ ± 1.29	111.25^a^ ± 2.16	< 0.01

Control= basal diet, SPP1 = basal diet + 0.1 g Spirulina/kg BW, SPP2 = basal diet + 0.2 g Spirulina /kg BW. Means ± SE represent least-squares means. Superscripts (a, b, c) within the same row indicate significant differences (*p* < 0.05, row-wise comparison)

**Table 8:** Fat-to-muscle-ratio (%) in lambs administered Spirulina

Time (days)	Treatments (mean ± SE)			
	Control	SPP1	SPP2	*p*-value
0	13.04 ± 0.67	13.23 ± 0.68	13.44 ± 0.81	0.95
30	14.62^b^ ± 1.03	17.21^ab^ ± 1.34	21.00^a^ ± 1.46	< 0.01
60	20.11^b^ ± 1.2	23.98^ab^ ± 2.05	25.86^a^ ± 1.01	< 0.01

Control= basal diet, SPP1 = basal diet + 0.1 g Spirulina/kg BW, SPP2 = basal diet + 0.2 g Spirulina /kg BW. Means ± SE represent least-squares means. Superscripts (a, b, ab) within the same row indicate significant differences (*p* < 0.05, row-wise comparison)

## Discussion

### Growth performance

The increased weight gain in Spirulina-treated lambs in this study is consistent with other studies on various breeds of sheep of Najdi (Alghonaim et al. 2022) and Awassi (Mahmud et al. 2025), and across multiple growth periods, including sucking (El-Deeb et al. 2023) and fattening periods (Hanafy 2023). The greater improvement in growth performance observed in the current study compared with previous reports may be attributed to oral drenching. Unlike prior studies that incorporated Spirulina into the diet, this method ensures precise, uniform intake for each lamb, enabling a more accurate assessment of Spirulina’s direct effects on growth performance. Even though Frafra lambs showed an insignificant increase in body weight (Mokhtar et al. 2023), the overall evidence from different breeds suggests that Spirulina supplementation may support growth performance in small ruminants under various production conditions.

Several mechanistic explanations can account for the improved growth in lambs administered Spirulina in the current study. Spirulina has the potential to raise the efficiency of nitrogen, antioxidant effects, and protein metabolism, which presents itself in the improved blood biochemical profile of the treated lambs, with the increase of protein status and the decrease of urea levels (Alghonaim et al. 2022; Hanafy 2023; Mokhtar et al. 2023; Mahmud et al. 2025). Besides, Spirulina is known as a bioactive nutritional additive that enhances digestive efficiency and ruminal anabolic activity (Spínola et al. 2024). Furthermore, Spirulina has favorable fermentation characteristics, particularly in the protein and polysaccharide fractions. This promotes ruminal microbial growth and fermentative activity, thereby increasing nutrient availability and contributing to growth (Wang et al. 2023).

It should be noted that lambs were fed in groups. Feed quantity was estimated based on body weight according to the NRC (2007) recommendations, and adjusted regularly to ensure equal nutrient provision across groups. Therefore, these improvements in growth performance may be attributed to nutrient utilization efficiency rather than to feed consumption.

From a production perspective, the observed improvements in lambs’ growth may suggest a trend toward higher feed efficiency and greater live-weight gain per unit of feed intake (Ellison et al. 2022). Although feed intake and feed conversion were not directly measured in this study, such responses could hypothetically contribute to reduced pressure on land and water resources and support more sustainable lamb production systems. Economically, the observed trends in growth performance could potentially be associated with shorter finishing periods and lower energy requirements, which might facilitate earlier market preparation and could enhance profitability, particularly in meat production systems (Yang et al. 2012).

### Rumen fermentation

The higher TVFAs in SPP2 lambs may indicate enhanced ruminal fermentation activity. Increased TVFA levels generally reflect greater carbohydrate fermentation, which may increase the availability of fermentative energy for growth and tissue deposition (Gressley et al. 2011). The present findings agree with those of El-Deeb et al. (2023), who found that the Spirulina supplementation in small ruminants increased feed digestion, microbial protein production, and fermentation activity. Wang et al. (2023) reported increased rumen papillae height, improved intestinal absorptive morphology, and elevated TVFA levels in Hu sheep fed Spirulina, thereby improving nutritional assimilation.

Moreover, the lower NH₃-N content in the rumen in the present study may suggest more efficient ammonia uptake by ruminal microorganisms for microbial protein synthesis, as reported by Wang et al. (2018). Reduced ruminal NH₃-N concentrations are generally considered indicative of improved nitrogen capture by rumen microbes, which may support more efficient nutrient utilization and ruminal fermentation dynamics. Such responses could improve digestive efficiency and increase energy availability for growth. These findings may therefore indicate a potential improvement in nitrogen utilization within the rumen. Improved nitrogen use efficiency in ruminants has also been discussed in relation to broader sustainability aspects of livestock production systems. In this context, some previous studies have suggested that enhanced rumen nitrogen utilization may be associated with lower methane emissions (El-Deeb et al. 2023; Choudhury et al. 2022). Further studies may help clarify the underlying microbial processes and the possible environmental implications, including their relationship with methane emissions.

### Blood biochemical and antioxidant responses

The observed increase in protein and albumin concentrations in SPP2 lambs may reflect improved protein nutritional status and could be associated with enhanced protein metabolism. Globulin levels in the SPP2 group were slightly higher, although not significantly different, indicating that any potential effects of Spirulina supplementation on immune function remain inconclusive. Monitoring these parameters may still provide useful insights as general indicators of protein and physiological status.

Additionally, the reduction in blood urea concentration in lambs receiving Spirulina may be related to decreased NH₃-N levels during ruminal fermentation, which could reduce their absorption into the blood. Lower nitrogen excretion may consequently contribute to a reduced metabolic load on the liver (Ghallab et al. 2016) and kidneys (Røjen et al. 2011). In the same group, reduced glucose levels may reflect the action of Spirulina’s bioactive components, as suggested by Ren et al. (2018). Similar trends have been reported in lambs fed microalgae-based diets, indicating potential improvements in metabolic homeostasis and the efficiency of anabolic processes (Mahmud et al. 2025).

Finally, the marked redox adaptation observed was suggested by a substantial increase in total antioxidant capacity (Moreira et al. 2017). The bioactive pigments present in Spirulina, including β-carotene, phenolic compounds, and phycocyanin, may help enhance the body’s antioxidant defenses. According to Hanafy (2023), Spirulina supplementation can stimulate antioxidant mechanisms, thereby supporting redox homeostasis during rapid growth in lambs.

### Non-invasive ultrasound body composition traits

Improvement in the ultrasound-estimated *longissimus dorsi* muscle area, depth, and length, backfat depth, and intramuscular fat content of Spirulina-administered lambs may be an indication of enhanced tissue accretion and trends toward improved body composition. These findings suggest that Spirulina may promote nutrient partitioning, contributing to relative increases in lean tissue and intramuscular fat, consistent with trends reported in growing Najdi lambs (Alghonaim et al. 2022).

The observed increases in muscle area and perimeter may reflect potential improvements in muscle protein efficiency and fiber hypertrophy, possibly associated with the digestibility and balanced amino acid profile of Spirulina proteins (Pootthachaya et al. 2023). Similarly, the increase in MCI in treated lambs may reflect deeper, more compact *longissimus dorsi* muscles, suggesting potential improvements in muscle growth and muscularity during the fattening period. These trends align with previous studies indicating that protein- and nutrient-rich dietary supplements can support improvements in muscle morphology and lean tissue deposition (Orman et al. 2008; Pimentel et al. 2023). Overall, the observed changes in MCI may suggest a positive trend in muscle compactness and finishing quality, potentially related to the bioactive components of Spirulina, such as phycocyanin and essential fatty acids (Podgórska-Kryszczuk 2024).

Furthermore, the increase in FMR over the fattening period, particularly in SPP2 lambs, appears to reflect coordinated deposition of both muscle and fat rather than excessive fat accumulation, as these lambs also showed higher weight gain and larger muscle area. This pattern may indicate potential improvements in nutrient utilization, supporting muscle growth while maintaining moderate fat coverage, in agreement with previous findings (Pimentel et al. 2023).

Additionally, the observed increase in IMF may indicate potential improvements in meat quality, given its positive association with meat tenderness and flavor (Frank et al. 2017). The responses of adipose and muscular tissues appear consistent with a balanced anabolic trend rather than excessive fat deposition, a characteristic that may contribute to an ideal carcass structure (Ramos-Romero et al. 2021).

The current ultrasound-derived measurements could provide valuable directional insights into muscle growth, fat deposition, and tissue development. These values are intended to indicate relative changes and trends among treatment groups, rather than representing absolute carcass measurements. Therefore, these ultrasound-based findings are indicative rather than definitive evidence of improved carcass composition and should be understood as trend-estimating rather than exact numeric outcomes, reflecting the direction and potential of tissue development under Spirulina administration.

### Limitations and future prospects

The current study provides valuable insights into the effects of Spirulina administration on the growth performance and body composition dynamics of lambs under real farming conditions. Nevertheless, the study had some limitations regarding its scope and design. Body composition was monitored using ultrasonography, a non-invasive technique. This technique was chosen for measurement and monitoring. The method was not validated against actual measurements of the lambs’ carcasses. This was to allow for the repeated measurement and monitoring of the same animals without compromising their welfare. Although ultrasonography has been widely validated in the literature as a reliable proxy for carcass characteristics, the absence of slaughter-based validation in the current experiment limits interpretation of the results as absolute carcass outcomes.

Furthermore, the experimental period in this study was limited to short-term responses; hence, the long-term effects of Spirulina on growth characteristics, body composition, and metabolic adaptations need to be further established.

Moreover, the study primarily focused on biological and physiological responses, while carcass characteristics, feed intake, and economic efficiency were not directly assessed. These variables, however, must be considered in future research to make a more comprehensive appraisal of the feasibility and financial viability of Spirulina supplementation in small ruminant production systems.

## Conclusion

Oral administration of Spirulina *(Arthrospira platensis)* at two levels (0.1 and 0.2 g/kg body weight) for 60 days improved growth performance, rumen fermentation parameters, and ultrasonographically assessed body composition traits in growing Barki lambs. The dose of 0.2 g/kg BW produced the most pronounced responses, including greater *longissimus dorsi* muscle area and depth, as well as moderate increases in fat deposition estimated by ultrasound. By integrating Spirulina administration with longitudinal ultrasonography, this study introduces a practical, welfare-oriented approach for in vivo monitoring of tissue development. The integration of nutritional and imaging frameworks may provide a viable model that supports productivity while maintaining animal welfare in small ruminant production systems.

## Data Availability

The data sheets are available upon request during publication of the current paper.
